# In silico analysis of virulence factors of *Streptococcus uberis* for a chimeric vaccine design

**DOI:** 10.1007/s40203-023-00181-1

**Published:** 2024-01-06

**Authors:** Çiğdem Yılmaz Çolak

**Affiliations:** TUBITAK, Marmara Research Center, Gebze, Kocaeli Türkiye

**Keywords:** Reverse vaccinology, Multi-epitope vaccine, *Streptococcus uberis*, Bovine mastitis

## Abstract

*Streptococcus uberis* is one of the causative agents of bovine mastitis, which has detrimental effects on animal health and the dairy industry. Despite decades of research, the requirement for effective vaccines against the disease remains unmet. The goal of this study was to create a multi-epitope vaccine using five virulence factors of *S*. *uberis* through the reverse vaccinology approach, which has been employed due to its high efficiency and applicability. Plasminogen activator A (PauA), glyceraldehyde-3-phosphate dehydrogenase C (GapC), C5a peptidase, *S*. *uberis* adhesion molecule (SUAM), and sortase A (SrtA) were selected for the T cytotoxic (CTL) and B cell epitope analyses as they were extensively studied in *S*. *uberis* or other pathogens. Eighteen CTL and ten B cell epitopes that were antigenic, non-toxic, and non-allergenic were selected in order to design a chimeric vaccine candidate that in silico analysis revealed to be potentially immunogenic, non-allergenic, and stable. Molecular docking analysis of the vaccine candidate with Toll-like receptor (TLR) 2 and TLR 4 revealed stable interactions between the candidate and the immune receptors. Meanwhile, the stability of the docked complexes was confirmed using normal mode analysis. Additionally, in silico immune simulation of the vaccine candidate demonstrated the stimulation of primary immune responses, indicating that the chimeric protein can hold promise as a viable vaccine candidate for preventing* S*. *uberis* mastitis. Moreover, the current study can provide a background for designing epitope-based vaccines based on the explored epitopes.

## Introduction

Bovine mastitis, characterized by inflammation of the mammary gland, is a common disease in dairy cattle, leading to significant economic losses by affecting milk quality and yield. Mastitis-associated risk factors in lactating and non-lactating cows include udder structure, genetic aspects, diet, age of cows, stage of lactation, milking system, milking interval, hygiene, and dry period (Zigo et al. 2021). The main cause of bovine mastitis is pathogens like *Staphylococcus aureus*, *Escherichia coli*, *Streptococcus uberis*, *S. agalactiae*, *S. dysgalactiae*, and *Klebsiella pneumoniae* (Fessia and Odierno 2021). The incidence of mastitis has decreased since the introduction of the mastitis five-point plan in the 1970s. This plan included i) dry cow therapy; ii) diagnosis and treatment of clinical cases; iii) culling chronic cases; iv) post-milking teat disinfection; and v) routine maintenance of milking machines. However, it resulted in a shift in the predominance of some clinical mastitis isolates, such as *E*. *coli* and *S*. *uberis* (Sherwin and Breen 2022). Thus, about 80% of mastitis cases occur due to *S*. *aureus, E. coli*, *S. uberis*, and *S. dysgalactiae*. *S*. *uberis* has been frequently encountered in infections in both lactating and non-lactating cows around the world. It can cause chronic infection of the mammary glands promoted by biofilm and capsules, which offer resistance to phagocytosis and intracellular destruction by leukocytes (Tabashiri et al. 2022). The genetic heterogeneity of the pathogen and its ability to retain itself on several sites, such as the skin, oral cavity, and respiratory tract, may greatly influence the efficacy of mastitis control programs (Rainard et al. 2021). Moreover, *S*. *uberis* strains have shown an increased trend in antibiotic resistance worldwide (Sherwin and Breen 2022). Therefore, new approaches are required to effectively combat this devastating pathogen.

Treatment of bovine mastitis mainly relies on the use of antibiotics; nevertheless, due to the nature of the pathogen, efforts should be focused on the prevention of the infection (Rainard et al. 2021). The first attempts to develop a vaccine against *S*. *uberis* used either live or killed bacteria, neither of which offered efficient protection against the disease caused by different strains of *S*. *uberis*. Although subunit vaccines including virulence factors of the pathogen have been developed, they have limited efficacy in preventing the disease (Kerro Dego et al. 2021). The only commercially available vaccine is UBAC® (Hipra, Spain), which consists of a biofilm adhesion component, lipoteichoic acid, from *S*. *uberis* strain 5616 (Collado et al. 2018). Although the vaccine can reduce infection severity and clinical mastitis cases, its effect on milk yield is small, and the disease cannot be fully prevented (Kabelitz et al. 2021). Despite the failure of previous attempts, studies are continuously being conducted to develop more efficient vaccines against *S*. *uberis* mastitis.

The fields of immunoinformatics and reverse vaccinology have witnessed significant advancements in line with our understanding of the host immune response. This progress has given rise to novel disciplines in computer-aided vaccine design, leveraging in silico epitope predictions. One promising approach in this field is the identification of specific epitopes derived from infectious agents, leading to the construction of epitope-driven vaccines (Gaafar et al. 2019; Forouharmehr et al. 2022; Kar et al. 2022). The epitope-based vaccine has many advantages over traditional vaccines, such as the ability to induce immune response without the risk of cytokine storms, accurate and rapid design, and cost-effective formulation (Parvizpour et al. 2020). In recent years, many studies have been implemented to design epitope-based vaccines against both human and animal pathogens using computational tools; some of these vaccines have been validated in the laboratory through animal studies (Forouharmehr et al. 2022; Rahimnahal et al., 2022; Samad et al. 2022). The study conducted by Majidiani et al. (2021), for example, revealed the protective capacity of a multi-epitope vaccine containing fifteen epitopes against *Toxoplasma gondii* in mice, and another study demonstrated the induction of strong humoral and cellular immune responses by a multi-epitope vaccine containing eleven non-redundant MHC I/II binding epitopes against *Mycoplasma hyopneumoniae* in mice and piglets (Li et al. 2022). Moreover, clinical trials have been conducted with some multi-epitope vaccines, including Multimeric-001 (M-001) and Flu-v, which are polyepitope vaccines consisting of epitopes from Influenza virus (Romeli et al. 2020).

Despite the increasing prevalence and significant economic losses associated with *S*. *uberis* mastitis, the virulence factors behind bacterial colonization and pathogenicity have not yet been fully understood. Over the past three decades, several virulence factors of *S*. *uberis* have been described, including plasminogen activator A (PauA), acid capsule, hyaluronidase, *S*. *uberis* adhesion molecule (SUAM), glyceraldehyde-3-phosphate dehydrogenase C (GapC), Opp proteins, elongation factor Ts, hemolysin-like protein, sortase A (SrtA), surface lipoprotein, fibronectin-binding protein, C5a peptidase, lactoferrin-binding protein, and collagen-like surface-anchored protein (Fessia and Odierno 2021). Among these factors, PauA, GapC, and SUAM have been extensively studied for the development of vaccines against *S*. *uberis* mastitis (Rainard et al. 2021). Moreover, the protective effects of C5a peptidase and SrtA were well documented against other *Streptococcus* sp., such as Group A and B Streptococci (Cleary et al. 2004; Gianfaldoni et al. 2009; Wang et al. 2019). Therefore, these five proteins were prioritized for the development of a multi-epitope vaccine against *S*. *uberis* mastitis.

In this study, the aim is to construct a multi-epitope vaccine candidate against *S*. *uberis* by employing a reverse vaccinology approach. In order to achieve this, PauA, GapC, C5a peptidase, SUAM, and SrtA were selected and analyzed to develop a chimeric vaccine design.

## Methodology

### Protein sequence collection

**Fig. 1:**
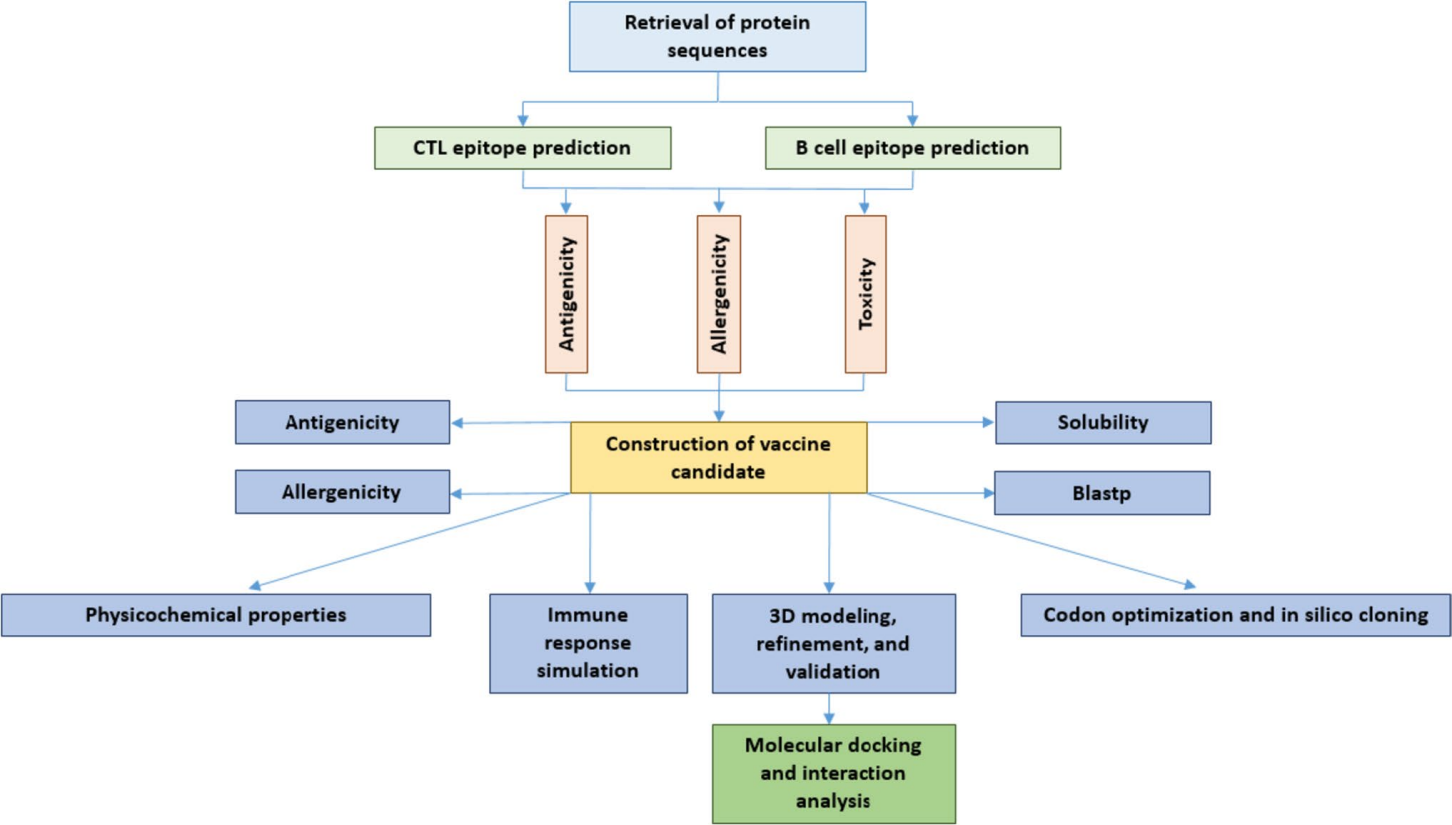
Graphical presentation of the study

The workflow diagram of the study was depicted in Fig. 1. In the study, *S*. *uberis* ATCC BAA-854/0140 J was selected as the reference strain, and five virulence factors of the strain, including PauA (B9DW72), GapC (B9DVS1), SrtA (B9DS55), SUAM (B9DVS5), and C5a peptidase (B9DSH4), were given priority for investigation. The complete sequences of all proteins were retrieved in FASTA format from the UniProt Database (https://www.uniprot.org/). This database is known for its provision of high-quality sequences accompanied by thorough annotation (UniProt Consortium 2023).

### Epitope selection

#### CTL epitope prediction

Cytotoxic (CTL) and helper (HTL) T cells play a crucial role in eliciting a robust immune response. Due to the limited availability of comprehensive characterization on major histocompatibility complex (MHC) class II molecules in buffalo and cattle, T cell epitope analysis was carried out using only bovine leukocyte antigen (BoLA) class I alleles, representing the MHC class I in bovine (Gaafar et al. 2019). The 9-mer CTL epitopes were predicted through the NetMHCpan EL 4.1 method in the Immune Epitope Database (IEDB) (https://www.iedb.org/). The selection threshold was based on the score and percentile rank; strong binders were indicated by a score above 0.5 and a percentile rank below 2%. The selected epitopes were subjected to the MHC class I immunogenicity analysis tool of IEDB (Vita et al. 2019).

#### Linear B cell epitope prediction

Linear B cell epitopes are short peptide sequences that play a critical role in initiating the humoral immune response. They are essential for combating pathogens through the production of specific immunoglobulins (Igs). The IEDB resource was used to predict linear B cell epitopes by employing Bepipred linear epitope prediction 2.0, and the epitopes between 6 and 20-mer were selected.

#### Antigenicity, allergenicity, and toxicity

All projected CTL and B cell epitopes were subjected to antigenicity analysis through the VaxiJen v2.0 tool (http://www.ddg-pharmfac.net/vaxijen/VaxiJen/VaxiJen.html) (Doytchinova and Flower 2007). The server houses a reference database of antigens and employs an auto-cross covariance (ACC) transformation method to assess the antigenicity of a protein based on its amino acid sequence. An epitope with a threshold of > 0.4 was considered a putative immunogen. The sequences less than 7-mer in length were not evaluated by VaxiJen, and thus 6-mer B cell epitopes were excluded from the analysis. The AllergenFP server (https://ddg-pharmfac.net/AllergenFP/index.html) employing a four-step differentiation approach with an accuracy of 88.9% was used to analyze allergenicity, and the epitopes with ‘non-allergen’ status were selected for further study (Dimitrov et al. 2014). Toxicity of the prioritized CTL and B cell epitopes was predicted by ToxinPred (http://crdd.osdd.net/raghava/toxinpred/) (Gupta et al., 2013).

### Multi-epitope vaccine candidate

#### Assembling of vaccine candidate and similarity analysis

The non-antigenic, allergenic, and toxic CTL and linear B cell epitopes were excluded, and the remaining epitopes were used to construct a chimeric vaccine candidate. First, CTL epitopes were aligned using AAY linkers between epitopes to promote epitope presentation and reduce junctional epitopes. Subsequently, B cell epitopes were introduced using KK linkers to enhance proteasome processing for epitope presentation (Tarrahimofrad et al. 2021; Sanami et al. 2022). To assess potential similarities, the final vaccine construct was subjected to NCBI BLASTp against the non-redundant protein sequence database of the bovine proteome.

#### Immonological and physiochemical properties

Similar to the epitopes, VaxiJen and AllergenFP were employed to assess the antigenicity and allergenicity of the vaccine construct, respectively. The ProtParam server (https://web.expasy.org/protparam/) was used to evaluate the physicochemical properties. This server provides information about the aliphatic index, isoelectric point (pI), molecular weight, the grand average of hydropathy, and instability index (Gasteiger et al. 2005). The solubility of the candidate was determined by Protein-Sol (https://protein-sol.manchester.ac.uk/) (Hebditch et al. 2017).

#### Secondary and tertiary structure prediction

The secondary structure of the vaccine construct was predicted using PDBsum (http://www.ebi.ac.uk/thornton-srv/databases/pdbsum/) (Laskowski et al. 2018). The tertiary structure of the construct was modeled using the trRosetta server (http://yanglab.nankai.edu.cn/trRosetta/) (Du et al. 2021).

The refinement of the 3D model was performed using GalaxyRefine (https://galaxy.seoklab.org/). This refinement entails the repackaging of protein side chains and their subsequent replacement with the most likely rotamers. By focusing on the protein core and extending towards the external surface, this refinement method improves the structural and functional stability of the protein. Consequently, it improves the quality of both local and global structures (Heo et al. 2013). The SAVES server (https://saves.mbi.ucla.edu/) was employed to analyze the statistics of non-bond interactions between different atoms within the predicted 3D model and to assess the quality of the model further (Laskowski et al. 1993). Finally, the evaluation of the overall quality of the model was performed using the ProSA web tool (https://prosa.services.came.sbg.ac.at/prosa.php). This tool generates a Z-score, which is used as a measure of quality (Wiederstein and Sippl 2007).

### Interaction of the construct with immune cell receptors

To ensure an effective immune response, it is crucial for an antigenic molecule to interact appropriately with specific immune receptors. Thus, protein–protein docking was employed to predict the interaction between the final vaccine construct and Toll-like receptor (TLR) 2 and TLR 4. The 3D structures of the extracellular domains of bovine TLR 2 and TLR 4 were modeled using a homology-based approach with the assistance of SWISS-MODEL (https://swissmodel.expasy.org/) (Waterhouse et al. 2018). The resulting models were further refined using GalaxyRefine (Heo et al. 2013). For docking studies, the ClusPro server (https://cluspro.bu.edu/home.php/) was used with default parameters by uploading PDB files of the vaccine model and the receptors, and interacting residues in the docked complex were demonstrated by the PDBsum server (Kozakov et al. 2017; Laskowski et al. 2018).

### Normal mode analysis

Normal mode analysis (NMA) involves the computational simulation of atomic and molecular motion, enabling their interactions to be studied within a defined period. This simulation provides valuable insights into the dynamic behavior and stability of the complex. The best-shortlisted vaccine construct underwent simulation using iMODS (https://imods.iqfr.csic.es/), which replicates a near-natural environment like a cell for proteins (López-Blanco et al. 2014). By means of this simulation, the most favorable poses of the proteins can contribute to the determination of their stability and potential effectiveness.

### Immune response simulation

In order to understand the immunogenicity and immune response characteristics of the vaccine construct, computer-based immune simulations were performed using C-ImmSim (https://kraken.iac.rm.cnr.it/C-IMMSIM/) (Rapin et al. 2010). The C-ImmSim server employs a position-specific scoring matrix (PSSM) for predicting immune epitopes and utilizes machine-learning techniques to predict immune interactions. The tool simultaneously simulates three compartments representing distinct anatomical regions found in mammals: (i) the thymus, (ii) the bone marrow, and (iii) a tertiary lymphatic organ, such as a lymph node. The simulation was carried out with three injections spaced four weeks apart, which is usually the recommended schedule for most vaccine practices. The parameters were kept at their default values, with time steps set at 1, 84, and 168, and simulation steps at 1000, where each time step corresponds to 8 h. So, time step 1 represents the injection at time = 0, while time step 84 represents 28 days, and time step 168 represents 56 days following the first dose (Pathak et al. 2022).

### Codon optimization and in silico cloning

Codon adaptation is employed to enhance the translation efficiency of foreign genes in a host organism, particularly when there are variations in codon usage between the host and the source of the gene. The codon optimization was performed using the Java Codon Adaptation Tool (JCAT) (http://www.jcat.de/), in which *E. coli* K12 was selected as the expression host (Grote et al. 2005). In order to achieve effective optimization, specific options were chosen to avoid potential issues. These included eliminating rho-independent transcription termination sites, prokaryotic ribosome binding sites, and restriction enzyme cleavage sites. Moreover, sticky end restriction sites for BamHI and HindIII were added to the sequence for ease of restriction and cloning. Subsequently, the modified sequence of the vaccine construct was cloned into the pET30a( +) vector using the SnapGene tool (https://snapgene.com/).

## Results

### Epitope selection

In cattle, the MHC molecules are referred to as BoLA molecules, which share structural and functional similarities with MHC molecules found in other mammals. The specific combination of BoLA alleles within an individual animal determines its MHC profile and influences its immune recognition and response capabilities (Takeshima and Aida 2006). Epitope prediction was carried out by the IEDB resource, consisting of more than 260,000 epitopes and over 1,200,000 T cell, B cell, MHC binding, and MHC ligand elution assays (Fleri et al. 2017).

#### CTL epitope

CTL cells are a crucial component of the immune system involved in cell-mediated immunity. They play a vital role in the defense against various pathogens, particularly intracellular pathogens such as viruses. They achieve this through the recognition of antigenic peptides presented by MHC I molecules on the surface of infected host cells (Thakur et al. 2019). In the study, BoLA-6*01301 (HD6), BoLA-2*01201 (T2A), BoLA-3*00201 (JSP.1), and BoLA-1*02301 (D18.4), BoLA-3*00101 (AW10), BoLA-6*04101 (T2B), BoLA-T2C, and BoLA-T5 were selected as the representative MHC I alleles of three bovine subspecies (*Bos taurus taurus*, *Bos taurus indicus*, and the hybrid *Bos taurus taurus x Bos taurus indicus)* (Hansen et al. 2014). For CTL epitopes, the IEDB analysis yields a list with the score and percentile rank; a lower percentile rank indicates higher binding affinity. The epitopes with scores higher than 0.5 and percentile ranks less than 2% were selected (Bahmani et al. 2021). These epitopes were subjected to immunogenicity analysis in IEDB, and immunogenic epitopes were further subjected to the VaxiJen server, where epitopes with scores below the default threshold of 0.4 were eliminated (Ali et al. 2017). Additionally, AllergenFP and ToxinPred servers identified non-allergenic and non-toxic epitopes, respectively (Table 1).

**Table 1:** Antigenic, non-allergenic, and non-toxic CTL and B cell epitopes predicted from the selected proteins of *S*. *uberis*

Protein	CTL epitope sequence	VaxiJen score	B cell epitope sequence	VaxiJen score
PauA	GEKHIGEKL	0.8116	None	–
GapC	EVNGNFIKV AIGLVIPEL	1.5860 0.7180	TTQGRFDGTVE AKKAAAEKHLHA ILDGPHRGGDLRRARA	1.0558 1.0303 0.9913
SrtA	AIPDVGINL AVSTDTVLK	1.2262 0.8577	None	–
SUAM	KLQGEEATL AIDIKTATL SQINVLTLK ELAVFAATL ALLGTGVGM DTAQEATIL	1.4655 1.4123 1.0538 0.5294 0.4232 0.4063	ENTKSNQIKTTLALTS	1.1525
C5a peptidase	DLSIPFIAF NQFELRVTL KIDGFSPEM ELRVTLHNL KVPASITLK KTDHFFFVR VGFRGVFLR	1.7980 1.7643 1.6109 1.3021 1.0461 0.5017 0.4303	SNQEKN LSVDGNM VYVTAADGLSKIQLGDVNNQ SNFDQQLKAQMPNG QKEYPHLSAKEQLQ VGKASHELND	1.4530 1.3931 0.6618 0.5886 0.5855 0.5399

##### B cell epitopes

B cells contribute to immune responses by producing antibodies and establishing immunological memories, which provide long-lasting protection against pathogens. According to the IEDB analysis, the B cell epitopes between 6 and 20-mer in length were prioritized since their length typically ranges from 5 to 20 amino acids (Lim et al. 2021). However, 6-mer B cell epitopes were excluded due to the minimum length limitation of the VaxiJen server. The same workflow of CTL epitopes was implemented, and antigenic, non-allergenic, and non-toxic linear B cell epitopes were specified (Table 1). However, no valid B cell epitope satisfying the specified criteria was obtained for PauA and SrtA proteins; therefore, they were not included in further analysis.

### Characterization of vaccine candidate

#### Vaccine engineering

**Fig. 2:**
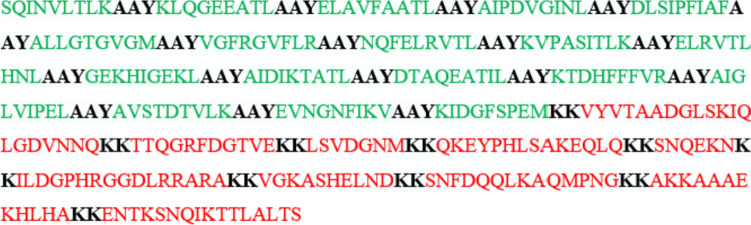
The amino acid sequence of the multi-epitope vaccine construct. CTL epitopes (green) were joined by AAY linkers, while linear B cell epitopes (red) were linked by KK residues

Since no HTL epitopes were assigned due to the lack of a complete characterization of bovine MHC II molecules, only CTL and B cell epitopes were used to construct a multi-epitope vaccine (Mollazadeh et al. 2022). Immunogenic, non-allergenic, antigenic, and non-toxic epitopes were selected among these epitopes because they should elicit an immune response without endangering the host. AAY and KK were used to conjugate the CTL and B cell epitopes, respectively. These linkers enhance protein stability through flexibility, facilitate protein folding, enable the separation of multiple domains, and promote epitope presentation (Dong et al. 2020; Tarrahimofrad et al. 2021; Sanami et al. 2022). Different combinations of the epitope sequences were analyzed, and the optimal combination was selected for further analysis. The final construct consisted of eighteen CTL epitopes and ten linear B cell epitopes, resulting in 359 amino acid residues in total (Fig. 2). The non-homologous, antigenic, and non-allergenic nature of the vaccine candidate was ascertained by performing the Blastp, VaxiJen, and AllergenFP analyses. The physicochemical properties of the final construct were presented in Table 2.

**Table 2:** Predicted physicochemical properties of the vaccine construct

Property	Result
Molecular weight	39.08 kDa
Solubility score	0.571
Theoretical isoelectric point (pI)	9.62
Instability index (II)	19.37
Grand average of hydropathicity (GRAVY)	– 0.220
Aliphatic index	90.45

#### Structural analysis of vaccine candidate

**Fig. 3:**
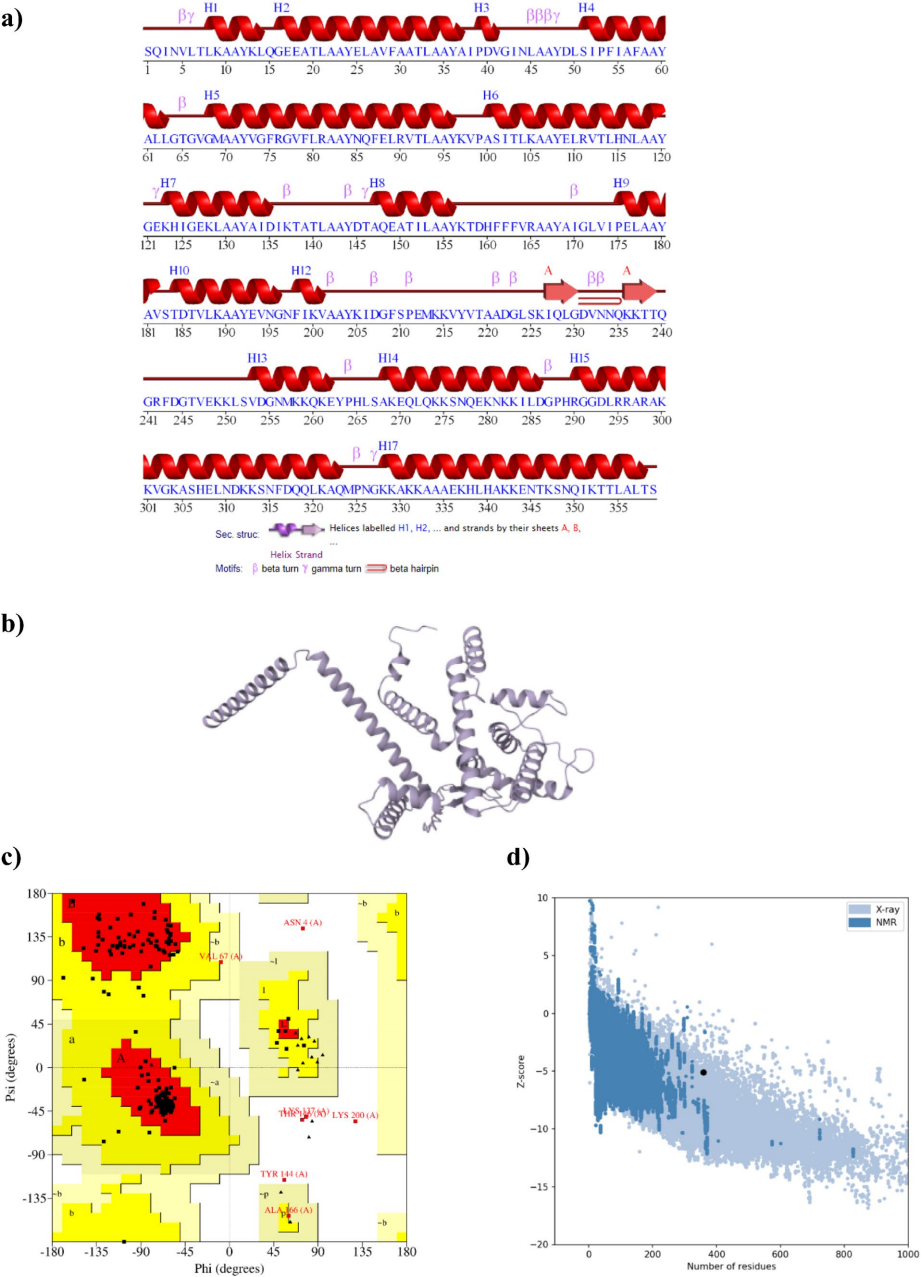
Design and structural characterization of the multi-epitope vaccine. **a** Secondary structure of the construct. **b** Three-dimensional model of the construct. **c** Ramachandran plot. **d** ProSA model quality

The pictorial prediction of the secondary structure was shown in Fig. 3a. The vaccine construct was composed of 17 helices, 18 beta turns, 5 gamma turns, 1 beta hairpin, and 2 strands. The 3D model of the vaccine construct was predicted using the trRosetta server, resulting in five models, and the first model was selected and subjected to further refinement. The Galaxyrefine tool provided five refined models of the construct, and model 1 (Fig. 3b) was chosen based on the parameters, including GDT-HA (0.9798), RMSD (0.328), MolProbity (1.698), clash score (6.7), poor rotamers (0.0), and Ramachandran plot (95.2) scores.

The refined model was further analyzed to evaluate the quality of the final vaccine construct. PROCHECK was utilized to generate a Ramachandran plot by evaluating the stereochemical quality of a protein structure by analyzing the geometry of individual residues and assessing the overall structure (Siddiki et al. 2023). The protein's Ramachandran plot indicated that the core region contained 91.7% of the amino acids, while 6.1% of the amino acids were situated in the allowed region. Additionally, 0.6% of the amino acids were found in the generously allowed region, and 1.5% of the amino acid residues were located in the disallowed region (Fig. 3c). The ERRAT program compares non-bonded interactions between various atom types in the protein with those observed in highly refined proteins. (Siddiki et al. 2023). Based on the ERRAT analysis, the overall quality factor of the vaccine construct was 91.86. In addition, ProSA provided a z-score of –5.11 (Fig. 3d).

### Molecular docking studies and simulations for stability

**Fig. 4:**
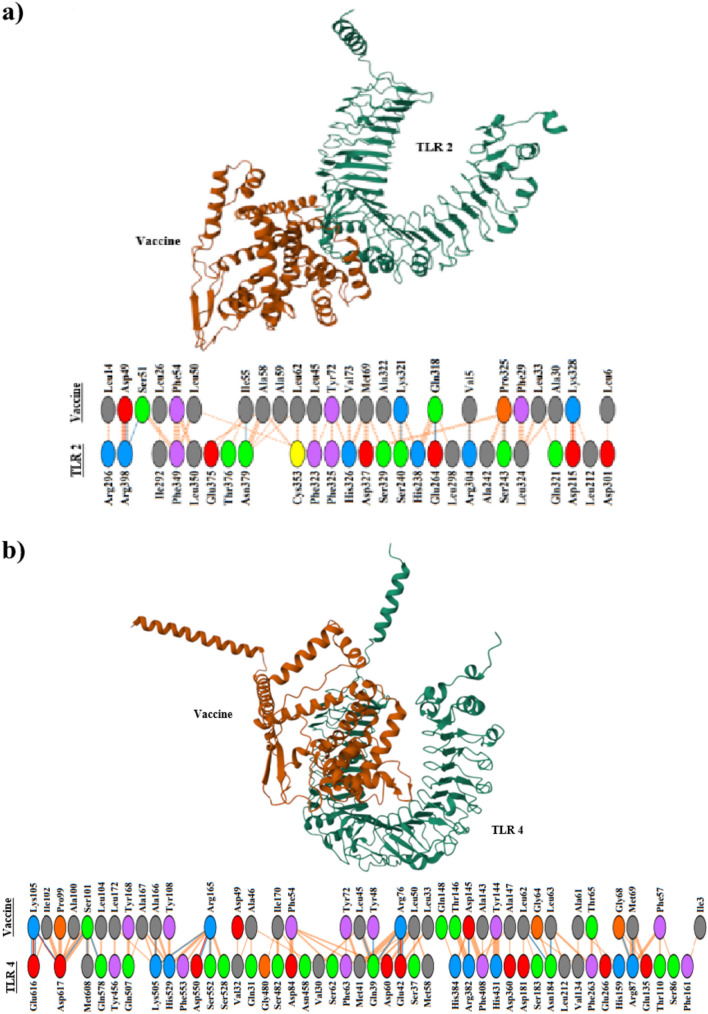
The binding conformation of the vaccine construct and TLR 2 (**a**) or TLR 4 (**b**). The detailed interactions between the receptors and the vaccine candidate were present below the docked complexes. Red line: salt bridges; yellow line: disulfide bonds; blue line: hydrogen bonds; striped line: non-bonded contacts. The width of the striped line was proportional to the number of atomic contacts

The docked complexes were generated by ClusPro, and the models with the lowest binding energy were selected for further analysis. The interactions between the construct and the receptors were graphically visualized by PDBsum. 2 salt bridges, 7 hydrogen bonds, and 115 non-bonded contacts were present between the vaccine construct and TLR 2 (Fig. 4a), while the vaccine-TLR 4 interaction had 4 salt bridges, 257 non-bonded contacts, and 20 hydrogen bonds (Fig. 4b), indicating more contact points between the construct and TLR 4.

**Fig. 5:**
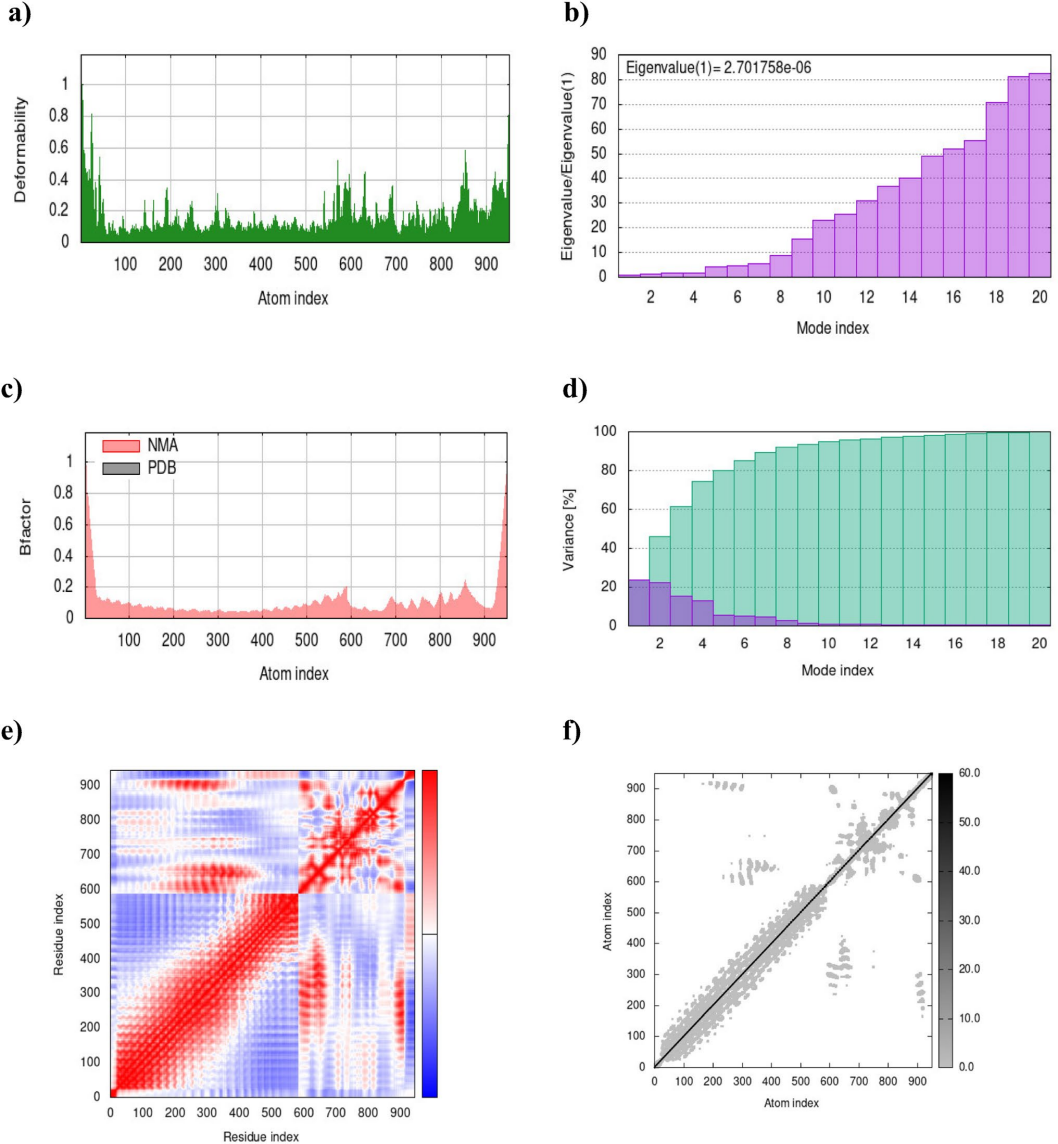
Stability and flexibility analysis of the vaccine-TLR 2 complex. **a** The main-chain deformability plot. **b** Eigenvalue plot. **c** B-factor plot. **d** Variance analysis. **e** Co-variance map. **f** Elastic network plot

**Fig. 6:**
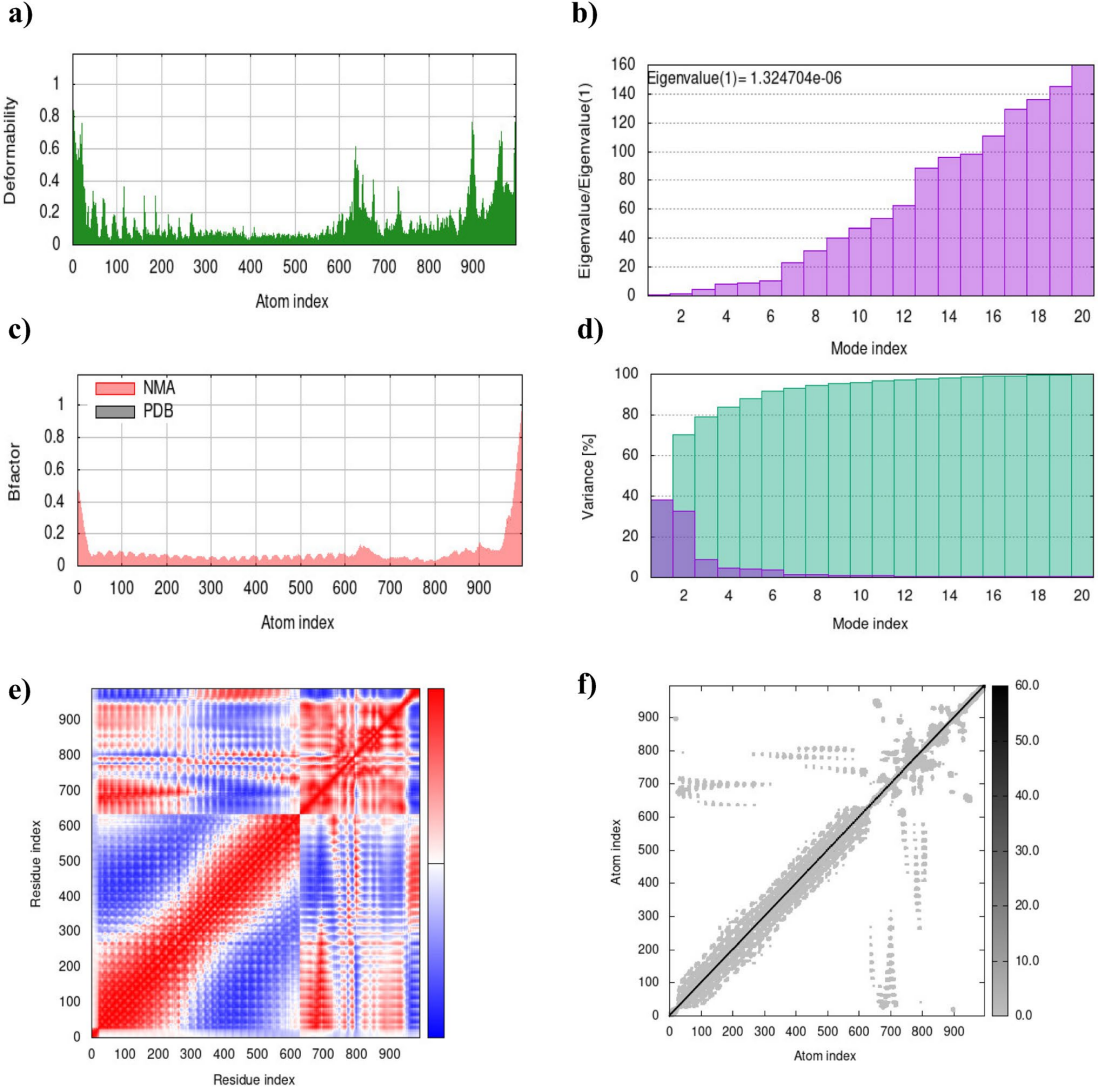
Stability and flexibility analysis of the vaccine-TLR 4 complex. **a** The main-chain deformability plot. **b** Eigenvalue plot. **c** B-factor plot. **d** Variance analysis. **e** Co-variance map. **f** Elastic network plot

The iMOD server utilizes NMA to assess the minimal deformation of the docked complex and evaluate its binding stability, which is demonstrated through various measures (López-Blanco et al. 2014; Zaib et al. 2023). The main-chain deformability plot indicates binding stability; lower deformability suggests a more stable interaction. The observed peaks in Figs. 5a and 6a were correlated to the deformed regions of the vaccine-TLR 2 and vaccine-TLR 4 complexes, respectively. The eigenvalue represents the amount of energy required to deform the docked complex, and a higher value indicates a greater energy barrier for deformation. The eigenvalues for the vaccine-TLR 2 and vaccine-TLR 4 complexes were 2.7e-06 and 1.3e-06, respectively, indicating considerable stability (Figs. 5b and 6b). The B-factor graph represents the average RMSD, illustrating how changes in the mobility of the complex relate to the scores obtained from PDB (Figs. 5c and 6c). The variance graph provides a better understanding of the distribution of motions within the complex. Cumulative variance is indicated by green bars, while purple bars indicate the variance of individual modes, and it is inversely related to the eigenvalue (Figs. 5d and 6d). Moreover, the covariance matrix highlights the coupling between residue pairs, where the uncorrelated, correlated, and anti-correlated motions are depicted in white, red, and blue colors, respectively. In the vaccine-TLR 4 complex, most of the regions were red, suggesting correlated motions of residues when compared to the vaccine-TLR 2 complex (Figs. 5e and 6e). The elastic network model provides insights into how the individual components within the complex move in response to external forces or ligand binding. In the elastic network plot, the darker gray dots indicate stiffer springs between atom pairs. According to the elastic models for the vaccine-TLR 2 (Fig. 5f) and vaccine-TLR 4 (Fig. 6f) complexes, the atoms up to 600 exhibited stronger spring formation.

### Immune simulation for vaccine efficacy

**Fig. 7:**
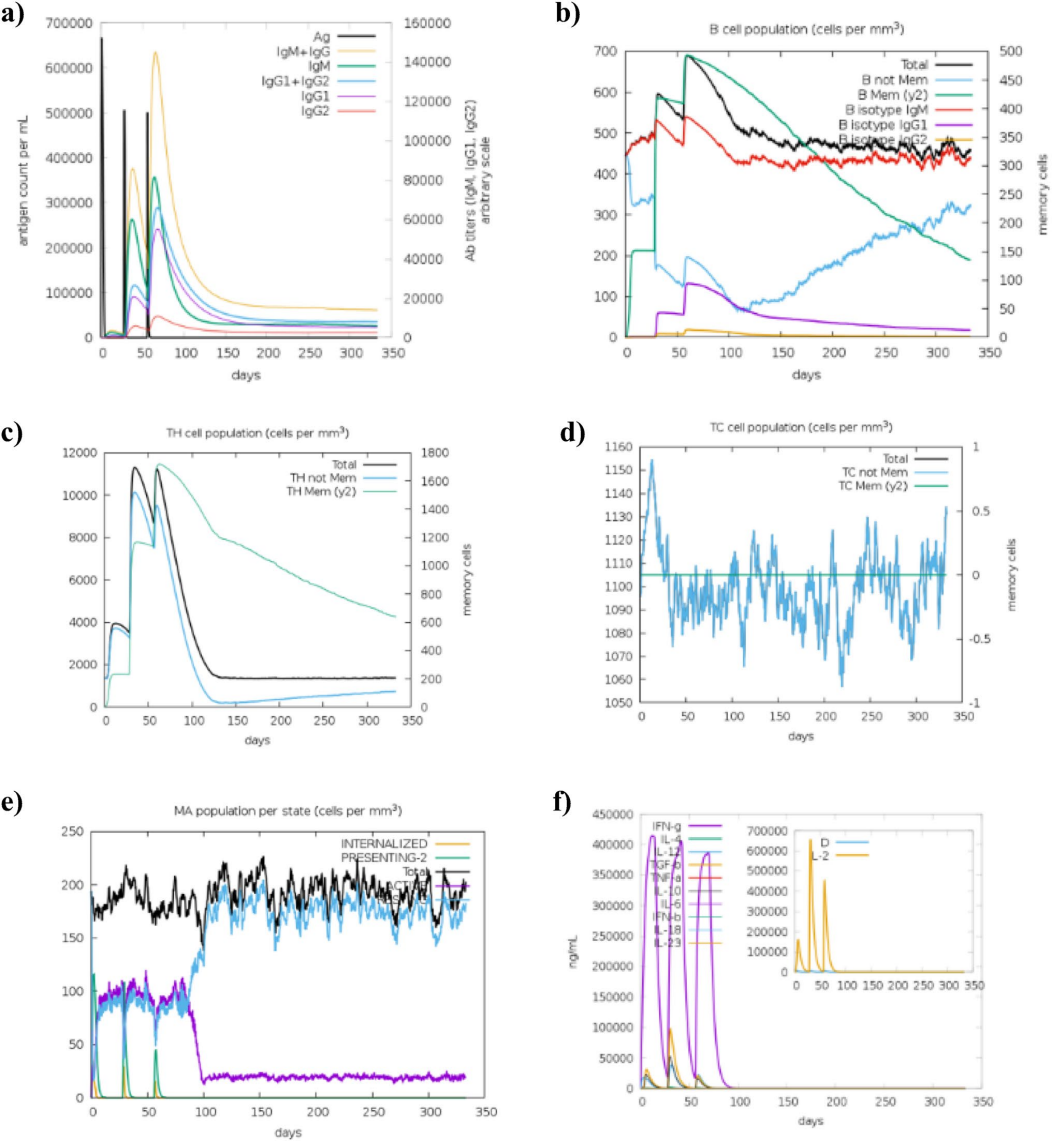
The results of immune response simulation with the vaccine construct. **a** Immunoglobulin production upon immunization. **b** The evolution of B cell populations. **c** Generation of helper T cells. **d** Generation of cytotoxic T cells. **e** Macrophage population per state. **f** Cytokine and interleukin production along with the Simpson index (d)

The immunogenic profile of the vaccine construct was depicted in Fig. 7. The administration of the vaccine resulted in an increase in the titers of antibodies, including IgM and IgG isotypes, which was followed by a further increase after the second and third immunizations (Fig. 7a). Also, a rise in B cell population was observed along with the different types of B cells, indicating their class-switching potential (Fig. 7b). Moreover, elevated levels of T helper (Fig. 7c) and cytotoxic T cell (Fig. 7d) populations were noticed after vaccine administration, suggesting the activation of cell-mediated responses. In addition, an increase in macrophage population per state was observed (Fig. 7e). Cytokine analysis revealed a considerable increase in the levels of different cytokines, especially interferon-gamma and interleukin-2, after immunizations (Fig. 7f). Moreover, a lesser Simpson index (D) indicated in Fig. 7f implied greater diversity in immune responses.

### Codon optimization and in silico cloning

**Fig. 8:**
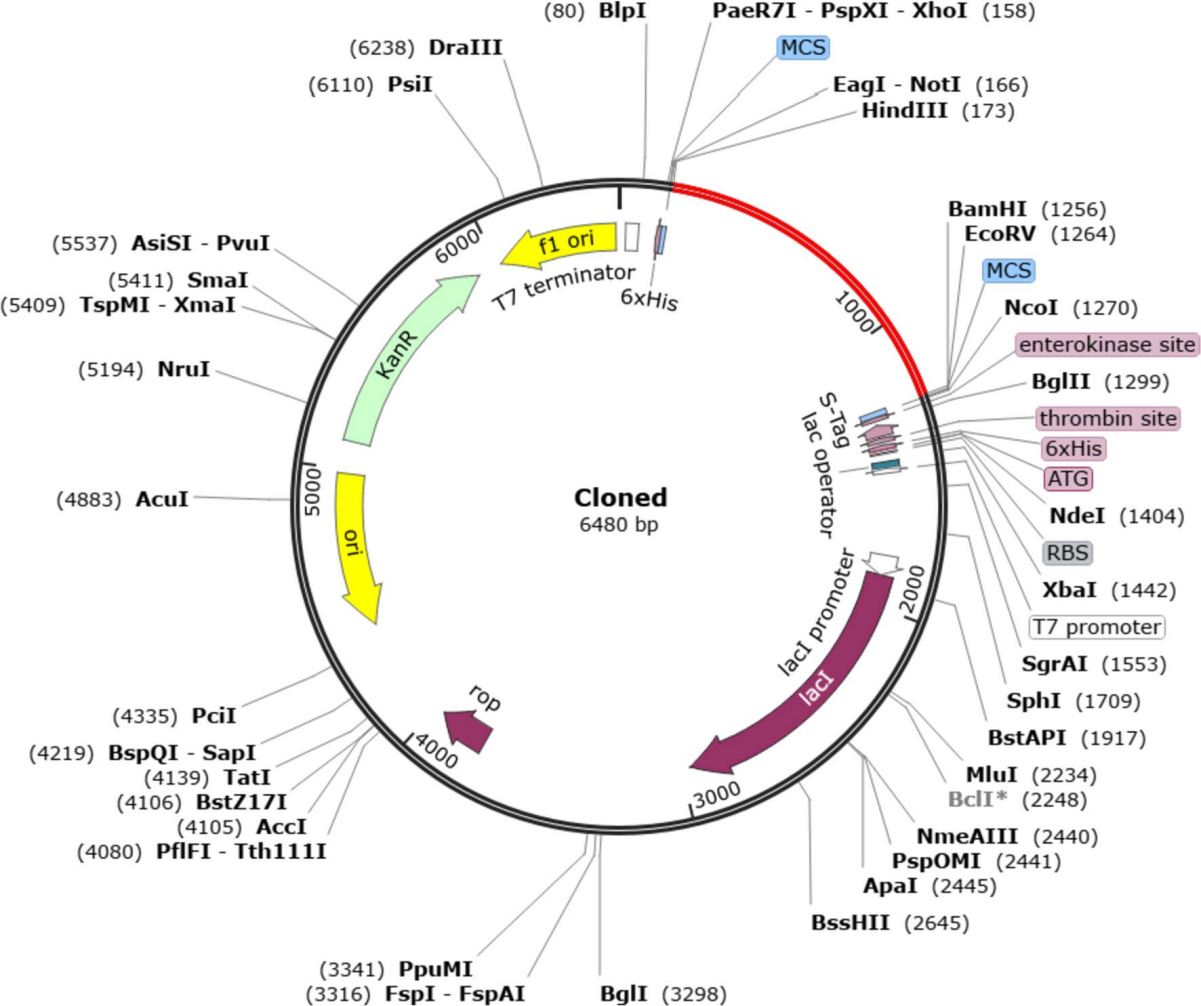
In silico cloning of the vaccine into pET30a(+) vector. The red fragment represents the insert

The JCat provides outputs of the Codon Adaptation Index (CAI) and the percentage of GC content, which play a crucial role in assessing protein expression levels. CAI indicates the biases in codon usage, and an ideal CAI score is 1.0, though scores above 0.8 are still considered acceptable. The GC content of a sequence should typically fall within the range of 30–70%, and values outside this range may indicate unfavorable effects on translational and transcriptional efficiencies (Ali et al. 2017). The optimized codon sequence consisted of 1,077 nucleotides. The CAI for this optimized sequence was 0.97, indicating a high likelihood of successful expression of the vaccine candidate in *E*. *coli* K12. Additionally, the average GC content of the adapted sequence was 48.46%, falling within the optimal percentage range, further supporting the potential for efficient expression. Finally, to construct the recombinant plasmid, restriction enzyme sites of HindIII and BamHI were added, and the codon sequence was inserted into the pET30a ( +) vector (Fig. 8).

## Discussion

Bovine mastitis is an inflammation of the mammary glands in dairy cattle that has been a concern for dairy farmers for decades. It affects milk production, milk quality, and the overall health of the dairy herd, resulting in economic losses in the dairy industry. The total cost of failure due to bovine mastitis is estimated at $147 per cow, with an annual global cost estimated to be between $19 billion and 32 billion (Cheng and Han 2020; Glasgow 2016). The primary causes of the cost are the following: milk production loss (31%), veterinary fees and drug costs (24%), premature culling (23%), and discarded milk (18%) (Rollin et al. 2015). The National Institute for Research in Dairying introduced a five-point plan to manage mastitis, but it hasn’t been able to completely eradicate the disease. Thus, it has been coupled with additional approaches such as improved hygiene practices and periodic monitoring to control the infection (Cheng and Han 2020).

*S*. *uberis* has been identified as one of the most prevalent environmental infectious agents in several countries, despite the fact that multiple bacterial species can cause bovine mastitis (Sherwin et al. 2021; Sherwin and Breen 2022). The transmission of *S*. *uberis* is mainly environmental, but within-cow and cow-to-cow transmission is also possible based on epidemiological studies. Moreover, the pathogen can easily adapt to a variety of niches, including the skin, oral cavity, feces, respiratory tract, and rectum, due to its nutritional flexibility (Fessia and Odierno 2021). The establishment of *S*. *uberis* mastitis involves the adherence to and internalization into mammary epithelial cells that provide protection against the immune system and non-specific antibacterial factors present in bovine milk (Almeida et al. 2015). *S*. *uberis* could survive intracellularly without causing apparent cell damage while sustaining a small population of viable pathogens, possibly serving as a source for persistent infections (Tamilselvam et al. 2006). Although the exact mechanism of the pathogenesis is still not clear, a number of virulence factors, including SUAM, PauA, SrtA, GapC, and C5a peptidase, have been suggested to play significant roles during the infection. SUAM, present on the cell surface, is one of the well-known virulence factors of *S*. *uberis*, and it has a central role in adherence and internalization events through its affinity for the host lactoferrin. PauA is secreted from *S*. *uberis*, and it degrades the extracellular matrix through the activation of plasminogen to plasmin, enhancing the invasion of the pathogen. Another virulence factor, SrtA, seems to anchor some proteins to peptidoglycan that are important for the establishment of infection, as SrtA mutants could not colonize in high numbers in the bovine mammary glands (Leigh et al. 2010). GapC is a surface dehydrogenase protein, having a possible role in virulence due to its ability to confer resistance against reactive oxygen species of the phagocytic cells (Zouharova et al. 2022). Lastly, C5a peptidase is a cell wall-anchored serine protease that cleaves the C5a component, resulting in the inhibition of bacterial opsonization and phagocyte recruitment (Fessia and Odierno 2021). All these virulence determinants have been investigated in terms of their protective capacities against *S*. *uberis* or other pathogens, and they can serve as potential vaccine candidates.

Many commercial vaccines contain a portion of pathogens, especially specific antigenic proteins, to trigger an immune response. All antigenic proteins may not always succeed as potential vaccines during their development due to: (a) the antigen not getting a proper immune response; (b) the antigen demonstrating weak antigenic or immunogenic properties; and (c) the antigen causing allergies in the host. However, thanks to recent advancements in in silico analysis, it is now feasible to pinpoint molecules with the highest potential to meet the criteria of a promising antigen (Kar and Srivastava 2018). The rapid development in bioinformatics and computational biology has fueled the creation of innovative therapeutic agents like multi-epitope vaccines, which are safe, stable, biologically harmless, and highly effective strategies against pathogens. The incorporation of computer-based methods provides a valuable tool to reduce laboratory-related costs and mitigate errors through the use of in silico approaches, given that experiments to identify antigenic determinants require large resources for consumables, materials, or ethical considerations with regard to animal use (Behbahani et al. 2021).

The current study, therefore, focused on the in silico design of a potential multi-epitope vaccine for bovine mastitis using five virulence factors expressed by *S*. *uberis*. First, CTL and B cell epitopes were predicted from the selected proteins, and the prioritized epitopes, including 18 CTL and 10 B cell epitopes, were fused using appropriate linkers to generate a multi-epitope vaccine candidate. The Blastp analysis revealed no homology with the bovine proteome, and VaxiJen provided a score of 0.8830, indicating the antigenicity of the candidate. Also, the non-allergenic nature of the candidate was revealed. The vaccine candidate was predicted to have a molecular weight (MW) of 39.08 kDa and a theoretical isoelectric point (pI) value of 9.62, indicating the basic nature of the protein. The predicted solubility score was 0.571, indicating that the protein was expected to be soluble upon expression. The candidate was classified as stable with an instability index (II) of 19.37 (II > 40 indicates instability). It also exhibited a predicted aliphatic index of 90.45, indicating potential thermostability at variable temperatures (Shey et al. 2019). The grand average of hydropathicity (GRAVY) was estimated to be -0.220, suggesting that the protein was hydrophilic and capable of interacting with water molecules (Forouharmehr et al. 2022). The 3D structure of the vaccine candidate was modeled using trRosetta, and its Ramachandran plot showed that most of the residues were present in the favoured and allowed regions (97.8%), with very few residues in the outlier region, indicating that the quality of the model was satisfactory (Kar et al. 2022). Based on the ERRAT analysis, the overall quality factor of the vaccine construct was 91.86, and ProSA provided a z-score of -5.11; these also indicated a favorable protein structure. The binding affinity of the construct to immune receptors, TLR 2 and TLR 4, was evaluated by performing ligand-receptor docking analysis, resulting in favorable interactions between the vaccine construct and the receptors. In addition, the stability of the interaction was verified using NMA, suggesting a stable interaction for both docked complexes. The immune response profile of the vaccine candidate was explored, and the results from the immune simulation study were in parallel with normal immune responses, including an increase in the levels of various cytokines and stimulation of cell-mediated responses.

Similar works have been performed for other pathogens causing bovine mastitis, such as *S*. *agalactiae*, *S. dysgalactiae*, and *Mycoplasma bovis* (Ali et al. 2021; Dzayee et al. 2021; Ma et al. 2021; Pathak et al. 2022). In these studies, researchers selected some antigenic proteins, namely Sip, GapC, and chromate transporter protein, from* S*. *agalactiae*, *S. dysgalactiae*, and *Mycoplasma bovis*, respectively, and revealed T and B cell epitopes of the proteins to construct multi-epitope vaccines. In general, the multi-epitope vaccines demonstrated stable interactions with immune receptors such as TLR 4, indicating their potential as viable vaccine candidates against these pathogens. Moreover, the vaccine construct derived from epitopes of GapC was experimentally evaluated, resulting in higher levels of IgG and cytokines, as well as a significant reduction in the colonization of S. *dysgalactiae* in the organs of immunized mice. This study provides optimism over the effectiveness of multi-epitope vaccines developed using in silico tools.

## Conclusion and limitations

Current approaches to control *S*. *uberis* mastitis in dairy farming, such as antibiotic therapy, teat disinfection, and culling of chronically infected animals, have shown limited efficacy in reducing the incidence and prevalence of the disease. To combat *S*. *uberis* infection effectively, new control measures, including the development of efficacious vaccines, are essential. In this study, a multi-epitope vaccine containing CTL and B cell epitopes from five virulence factors of *S*. *uberis* was designed using various immunoinformatics tools. Based on the outcomes, the vaccine candidate may have the potential to be effective in eradicating *S*. *uberis* infection, offering a promising approach to control mastitis in dairy farming. Therefore, the vaccine candidate can be an important starting point for developing a potent vaccine against mastitis through the expression of the protein in an appropriate host-vector system. Moreover, the epitopes identified in the study can be used in further studies.

Despite being a promising vaccine candidate, multi-epitope vaccines have low immunogenicity and are prone to enzymatic degradation. However, using proper adjuvants and carriers, like nanoparticles, can overcome these challenges (Machimbirike et al. 2022). Besides these general limitations of epitope vaccines, the study has a few shortcomings. It does not perform comprehensive experimental testing to confirm the effective fusion of epitopes in the vaccine construct. The study relies on predictions generated by servers, which may not be very accurate since these tools lack robust training data from real experiments. Additionally, in vitro and in vivo studies are necessary to evaluate the actual protective capacity of the vaccine candidate.

## References

[CR1] Ali M, Pandey RK, Khatoon N, Narula A, Mishra A, Prajapati VK (2017). Exploring dengue genome to construct a multi-epitope based subunit vaccine by utilizing immunoinformatics approach to battle against dengue infection. Sci Rep.

[CR2] Ali I, Shoukat T, Parveen T, Raza S, Jamil F, Kanwal S, Ibrahim M, Rasheed MA (2021) Multi epitope based vaccine desing and analysis against *Mycoplasma bovis* using immunoinformatic appraches. Pak Vet J 42(1):33–40. 10.29261/pakvetj/2021.068

[CR3] Almeida RA, Kerro-Dego O, Headrick SI, Lewis MJ, Oliver SP (2015). Role of *Streptococcus uberis* adhesion molecule in the pathogenesis of *Streptococcus uberis* mastitis. Vet Microbiol.

[CR4] Bahmani B, Amini-bayat Z, Ranjbar MM (2021). HPV16-E7 protein T cell epitope prediction and global therapeutic peptide vaccine design based on human leukocyte antigen frequency. Int J Pept Res Ther.

[CR5] Behbahani M, Moradi M, Mohabatkar H (2021). In silico design of a multi-epitope peptide construct as a potential vaccine candidate for Influenza A based on neuraminidase protein. In Silico Pharmacol.

[CR6] Cheng WN, Han SG (2020). Bovine mastitis: risk factors, therapeutic strategies, and alternative treatments – A review. Asian-Australas J Anim Sci.

[CR7] Cleary PP, Matsuka YV, Huynh T, Lam H, Olmsted SB (2004). Immunization with C5a peptidase from either group A or B Streptococci enhances clearance of group A streptococci from intranasally infected mice. Vaccine.

[CR8] Collado R, Montbrau C, Sitjà M, Prenafeta A (2018). Study of the efficacy of a *Streptococcus uberis* mastitis vaccine against an experimental intramammary infection with a heterologous strain in dairy cows. J Dairy Sci.

[CR9] Dimitrov I, Naneva L, Doytchinova I, Bangov I (2014). AllergenFP: allergenicity prediction by descriptor fingerprints. Bioinformatics.

[CR10] Dong R, Chu Z, Yu F, Zha Y (2020). Contriving multi-epitope subunit of vaccine for COVID-19: immunoinformatics approaches. Front Immunol.

[CR11] Doytchinova IA, Flower DR (2007). VaxiJen: a server for prediction of protective antigens, tumour antigens and subunit vaccines. BMC Bioinform.

[CR12] Du Z, Su H, Wang W, Ye L, Wei H, Peng Z, Anishchenko I, Baker D, Yang J (2021). The trRosetta server for fast and accurate protein structure prediction. Nat Protoc.

[CR13] Dzayee SA, Khudhur PK, Mahmood A, Markov A, Maseleno A, Gorji AE (2021). Computational design of a new multi-epitope vaccine using immunoinformatics approach against mastitis disease. Anim Biotechnol.

[CR14] Fessia AS, Odierno LM (2021). Potential factors involved in the early pathogenesis of *Streptococcus uberis* mastitis: a review. Folia Microbiol.

[CR15] Fleri W, Paul S, Dhanda SK, Mahajan S, Xu X, Peters B, Sette A (2017). The immune epitope database and analysis resource in epitope discovery and synthetic vaccine design. Front Immunol.

[CR16] Forouharmehr A, Nazifi N, Mousavi SM, Jaydari A (2022). Designing an efficient epitope-based vaccine conjugated with a molecular adjuvant against bovine babesiosis: A computational study. Process Biochem.

[CR17] Gaafar B, Ali SA, Abd-Elrahman KA, Almofti YA (2019). Immunoinformatics approach for multiepitope vaccine prediction from H, M, F, and N proteins of Peste des Petits ruminants virus. J Immunol Res.

[CR18] Gasteiger E, Hoogland C, Gattiker A, Wilkins MR, Appel RD, Bairoch A, Walker JM (2005). Protein identification and analysis tools on the ExPASy server. The Proteomics Protocols Handbook.

[CR19] Gianfaldoni C, Maccari S, Pancotto L, Rossi G, Hilleringmann M, Pansegrau W, Sinisi A, Moschioni M, Masignani V, Rappuoli R, Del Giugice G, Ruggiero P (2009). Sortase A confers protection against *Streptococcus pneumoniae* in mice. Infect Immun.

[CR20] Glasgow, U (2016) Potential biomarkers of mastitis in dairy milk identified. University of Glasgow.

[CR21] Grote A, Hiller K, Scheer M, Münch R, Nörtemann B, Hempel DC, Jahn D (2005). JCat: a novel tool to adapt codon usage of a target gene to its potential expression host. Nucleic Acids Res.

[CR22] Gupta S, Kapoor P, Chaudhary K, Gautam A, Kumar R, Open Source Drug Discovery Consortium, Raghava GP (2013). In silico approach for predicting toxicity of peptides and proteins. PLoS ONE.

[CR23] Hansen AM, Rasmussen M, Svitek N, Harndahl M, Golde WT, Barlow J, Nene V, Buus S, Nielsen M (2014). Characterization of binding specificities of bovine leucocyte class I molecules: impacts for rational epitope discovery. Immunogenetics.

[CR24] Hebditch M, Carballo-Amador MA, Charonis S, Curtis R, Warwicker J (2017). Protein–Sol: A web tool for predicting protein solubility from sequence. Bioinformatics.

[CR25] Heo L, Park H, Seok C (2013). GalaxyRefine: Protein structure refinement driven by side-chain repacking. Nucleic Acids Res.

[CR26] Kabelitz T, Aubry E, van Vorst K, Amon T, Fulde M (2021) The role of *Streptococcus* spp. in bovine mastitis. Microorganisms 9(7):1497. 10.3390/microorganisms9071497 10.3390/microorganisms9071497 PMC8305581 34361932

[CR27] Kar PP, Srivastava A (2018). Immuno-informatics analysis to identify novel vaccine candidates and design of a multi-epitope based vaccine candidate against *Theileria* parasites. Front Immunol.

[CR28] Kar PP, Araveti PB, Kuriakose A, Srivastava A (2022). Design of a multi-epitope protein as a subunit vaccine against lumpy skin disease using an immunoinformatics approach. Sci Rep.

[CR29] Kerro Dego O, Almeida R, Ivey S, Agga GE (2021) Evaluation of *Streptococcus uberis* surface proteins as vaccine antigens to control *S*. *uberis* mastitis in dairy cows. Vaccines 9(8):868. 10.3390/vaccines9080868 10.3390/vaccines9080868 PMC8402608 34451993

[CR30] Kozakov D, Hall DR, Xia B, Porter KA, Padhorny D, Yueh C, Beglov D, Vajda S (2017). The ClusPro web server for protein-protein docking. Nat Protoc.

[CR31] Laskowski RA, MacArthur MW, Moss DS, Thornton JM (1993). PROCHECK—a program to check the stereochemical quality of protein structures. J Appl Cryst.

[CR32] Laskowski RA, Jabłońska J, Pravda L, Vařeková RS, Thornton JM (2018). PDBsum: structural summaries of PDB entries. Protein Sci.

[CR33] Leigh JA, Egan SA, Ward PN, Field TR, Coffey TJ (2010). Sortase anchored proteins of *Streptococcus uberis* play major roles in the pathogenesis of bovine mastitis in dairy cattle. Vet Res.

[CR34] Li G, Shu J, Jin J, Shu J, Feng H, Chen J, He Y (2022). Development of a multi-epitope vaccine for *Mycoplasma hyopneumoniae* and evaluation of its immune responses in mice and piglets. Int J Mol Sci.

[CR35] Lim HX, Lim J, Jazayeri SD, Poppema S, Poh CL (2021). Development of multi-epitope peptide-based vaccines against SARS-CoV-2. Biomed J.

[CR36] López-Blanco JR, Aliaga JI, Quintana-Ortí ES, Chacón P (2014). iMODS: Internal coordinates normal mode analysis server. Nucleic Acids Res.

[CR37] Ma J, Wang L, Fan Z, Liu S, Wang X, Wang R, Chen J, Xiao X, Yang S, Duan X, Son B, Ma J, Tong Y, Yu L, Yu Y, Cui Y (2021). Immunogenicity of multi-epitope vaccines composed of epitopes from *Streptococcus dysgalactiae* GapC. Res Vet Sci.

[CR38] Machimbirike VI, Pornputtapong N, Senapin S, Wangkahart E, Srisapoome P, Khunrae P, Rattanarojpong T (2022). A multi-epitope chimeric protein elicited a strong antibody response and partial protection against *Edwardsiella ictaluri* in Nile tilapia. J Fish Dis.

[CR39] Majidiani H, Dalimi A, Ghaffarifar F, Pirestani M (2022). Multi-epitope vaccine expressed in *Leishmania tarentolae* confers protective immunity to *Toxoplasma gondii* in BALB/c mice. Microb Pathog.

[CR40] Mollazadeh S, Bakhshesh M, Keyvanfar H, Brujeni GN (2022). Identification of cytotoxic T lymphocyte (CTL) epitope and design of an immunogenic multi-epitope of Bovine Ephemeral Fever Virus (BEFV) glycoprotein G for vaccine development. Res Vet Sci.

[CR41] Parvizpour S, Pourseif MM, Razmara J, Rafi MA, Omidi Y (2020). Epitope-based vaccine design: a comprehensive overview of bioinformatics approaches. Drug Discov Today.

[CR42] Pathak RK, Lim B, Kim DY, Kim JM (2022). Designing multi-epitope-based vaccine targeting surface immunogenic protein of *Streptococcus agalactiae* using immunoinformatics to control mastitis in dairy cattle. BMC Vet Res.

[CR43] Rahimnahal S, Yousefizadeh S, Mohammadi Y (2023) Novel multi-epitope vaccine against bovine brucellosis: approach from immunoinformatics to expression. J Biomol Struct Dyn 1–25. 10.1080/07391102.2023.2188962 10.1080/07391102.2023.2188962 36927475

[CR44] Rainard P, Gilbert FB, Germon P, Foucras G (2021). Invited review: a critical appraisal of mastitis vaccines for dairy cows. J Dairy Sci.

[CR45] Rapin N, Lund O, Bernaschi M, Castiglione F (2010). Computational immunology meets bioinformatics: the use of prediction tools for molecular binding in the simulation of the immune system. PLoS ONE.

[CR46] Rollin E, Dhuyvetter KC, Overton MW (2015). The cost of clinical mastitis in the first 30 days of lactation: An economic modeling tool. Prev Vet Med.

[CR47] Romeli S, Hassan SS, Yap WB (2020) Multi-epitope peptide-based and Vaccinia-based universal influenza vaccine candidates subjected to clinical trials. Malays J Med Sci 27(2):10–20. 10.21315/mjms2020.27.2.2 10.21315/mjms2020.27.2.2 PMC7409566 32788837

[CR48] Samad A, Meghla NS, Nain Z, Karpiński TM, Rahman MS (2022). Immune epitopes identification and designing of a multi-epitope vaccine against bovine leukemia virus: a molecular dynamics and immune simulation approaches. Cancer Immunol Immunother.

[CR49] Sanami S, Rafieian-Kopaei M, Dehkordi KA, Pazoki-Toroudi H, Azadegan-Dehkordi F, Mobini GR, Alizadeh M, Nezhad MS, Ghasemi-Dehnoo M, Bagheri N (2022). In silico desing of a multi-epitope vaccine against HPV16/18. BMC Bioinform.

[CR50] Sherwin G, Breen J (2022). *Streptococcus uberis*-associated mastitis in dairy herds: dealing with outbreaks and improving control. In Pract.

[CR51] Sherwin VE, Green MJ, Leigh JA, Egan SA (2021). Assessment of the prevalence of *Streptococcus uberis* in dairy cow feces and implications for head health. J Dairy Sci.

[CR52] Shey RA, Ghogomu SM, Esoh KK, Nebangwa ND, Shintouo CM, Nongley NF, Asa BF, Ngale FN, Vanhamme L, Souopgui J (2019). In-silico design of a multi-epitope vaccine candidate against onchocerciasis and related filarial diseases. Sci Rep.

[CR53] Siddiki AZ, Alam S, Tithi FA, Hoque SF, Sajib EH, Hossen FFB, Hossain MA (2023). Construction of a multi-epitope in silico vaccine against *Anaplasma Marginale* using immunoinformatics approach. Biocatal Agric Biotechnol.

[CR54] Tabashiri R, Sharifi S, Pakdel A, Bakhtiarizadeh MR, Pakdel MH, Tahmasebi A, Hercus C (2022). Genome-wide post-transcriptional regulation of bovine mammary gland response to *Streptococcus uberis*. J Appl Genetics.

[CR55] Takeshima SN, Aida Y (2006). Structure, function and disease susceptibility of the bovine major histocompatibility complex. Anim Sci J.

[CR56] Tamilselvam B, Almeida RA, Dunlap JR, Oliver SP (2006). *Streptococcus uberis* internalizes and persists in bovine mammary epithelial cells. Microb Pathog.

[CR57] Tarrahimofrad H, Rahimnahal S, Zamani J (2021). Designing a multi-epitope vaccine to provoke the robust immune response against influenza A H7N9. Sci Rep.

[CR58] Thakur A, Mikkelsen H, Jungersen G (2019). Intracellular pathogens: host immunity and microbial persistence strategies. J Immunol Res.

[CR59] UniProt Consortium (2023). UniProt: The universal protein knowledgebase in 2023. Nucleic Acids Res.

[CR60] Vita R, Mahajan S, Overton JA, Dhanda SK, Martini S, Cantrell JR, Wheeler DK, Sette A, Peters B (2019). The Immune Epitope Database (IEDB): 2018 update. Nucleic Acids Res.

[CR61] Wang S, Zhao Y, Wang G, Feng S, Guo Z, Gu G (2019). Group A Streptococcus cell wall oligosaccharide-streptococcal C5a peptidase conjugates as effective antibacterial vaccines. ACS Infect Dis.

[CR62] Waterhouse A, Bertoni M, Bienert S, Studer G, Tauriello G, Gumienny R, Heer FT, de Beer TAP, Rempfer C, Bordoli L, Lepore R, Schwede T (2018). SWISS-MODEL: homology modelling of protein structures and complexes. Nucleic Acids Res.

[CR63] Wiederstein M, Sippl MJ (2007). ProSA-web: interactive web service for the recognition of errors in three-dimensional structures of proteins. Nucleic Acids Res.

[CR64] Zaib S, Rana N, Hussain N, Alrbyawi H, Dera AA, Khan I, Khalid M, Khan A, Al-Harrasi A (2023). Designing multi-epitope monkeypox virus-specific vaccine using immunoinformatics approach. J Infect Public Health.

[CR65] Zigo F, Vasil M, Ondrasovicova S, Vyrostkova J, Bujok J, Pecka-Kielb E (2021). Maintaining optimal mammary gland health and prevention of mastitis. Front Vet Sci.

[CR66] Zouharova M, Nedbalcova K, Slama P, Bzdil J, Masarikova M, Matiasovic J (2022). Occurrence of virulence-associated genes in *Streptococcus uberis* and *Streptococcus parauberis* isolated from bovine mastitis. Vet Med.

